# Social Cognition in a Clinical Sample of Personality Disorder Patients

**DOI:** 10.3389/fpsyt.2015.00075

**Published:** 2015-05-26

**Authors:** Amparo Ruiz-Tagle, Elsa Costanzo, Delfina De Achával, Salvador Guinjoan

**Affiliations:** ^1^Laboratorio de Nuerociencias Cognitivas, Centro de Investigación Avanzada en Educación, Universidad de Chile, Santiago, Chile; ^2^Department of Psychiatry, Fundación para la Lucha contra las Enfermedades Neurológicas de la Infancia (FLENI), Buenos Aires, Argentina

**Keywords:** social cognition, personality disorders, empathy, emotion recognition, theory of mind

## Abstract

Social cognition was assessed in a clinical sample of personality disorder (PD) stable patients receiving ambulatory treatment (*N* = 17) and healthy matched controls (*N* = 17) using tests of recognition of emotions in faces and eyes, in a test of social faux pas and in theory of mind (ToM) stories. Results indicated that when compared with healthy controls, individuals with PD showed a clear tendency to obtain lower scoring in tasks assessing recognition of emotion in faces (*T* = −2.602, *p* = 0.014), eyes (*T* = −3.593, *p* = 0.001), ToM stories (*T* = −4.706, *p* = 0.000), and Faux pas (*T* = −2.227, *p* = 0.035). In the present pilot study, PD individuals with a normal cognitive efficiency showed an impaired performance at social cognition assessment including emotion recognition and ToM.

## Introduction

Social cognition is the process that involves the mental operations that underlie behavior and social interaction; a specific cognitive domain, humans utilize to respond to social problems (1). The expression and recognition of emotions play an important role in social cognition (2), since the expressed emotions when identified by others, give clues about an individual’s mental state. Moreover, in order to interpret social signals in others, individuals must be capable of, on the one hand, empathy. Second, they must be able to utilize a reference framework that contains theories regarding the behavior of others, i.e., with regard to the thoughts and emotions that lead them to perform certain behaviors (3, 4). This is called theory of mind (ToM).

It has been observed that various areas of the brain are involved in social cognition; the sensory cortex is involved in the perceptual representation of stimuli and its characteristics; namely facial, postural, and emotional expressions as well as in movements with social signals (5, 6).

The emotional evaluation of stimuli humans perceive is processed by the amygdala; particularly as regards threat determination. Threats result in anxiety signals, which trigger an (autonomic) somatic response. In this sense, the amygdala is essential to interpreting social cues because it supplies emotional valuation and receives hippocampal projections that place them into context. In the case of antisocial subjects and individuals with social phobias, an inverse relationship exists between the excitability of the amygdala and the prefrontal cortex in response to social stimuli. While in antisocial individuals a hypoarousal is observed, a hyperactivation occurs in cases of social phobia (5).

The prefrontal cortex, orbitofrontal cortex, and particularly the ventromedial prefrontal cortex play similar roles in social behavior and are the most important regions in terms of social cognition tasks, decision-making, and social reasoning (2, 6). Changes such as decreased behavioral inhibition, lack of empathy, and incurrence of socially inappropriate behaviors due to orbitofrontal lesions are included. Apathy, akinesia, difficulty in initiating action, inappropriate social behavior, and changes in the pragmatics of language have been documented as outcomes of medial prefrontal lesions (7, 8). Neuroimaging studies have demonstrated left medial activation (ventral more than dorsal) for ToM tasks (2).

Most studies suggesting a link between social cognition and personality disorder (PD) are primarily based on deficits of this function in schizotypal and antisocial subjects given the inherent characteristics of both disorders. These attributes in turn explain why these subjects have low frequency of attendance at mental health centers. They also explain why in related studies (9–12) have been involved populations of subclinical students or inmates using personality questionnaires as the sole diagnostic tool for inclusion and exclusion of the sample.

In general, there cannot be found significant differences in simple ToM tasks (such as the recognition of emotions via facial expressions, or first and second-order false-belief) when comparing subjects who score high on schizotypy or psychopathy, with those who score low, whereas differences in more complex social cognition tasks have been described (13, 14). In addition, studies that correlate dysfunction in areas of the brain (orbitofrontal cortex, ventromedial, and amygdala primarily) with high impulsivity in patients with Cluster B under stress in Gambling and Go/No-Go tasks have been performed (15, 16). The same areas of the brain where the functions of social cognition reside would present a deficit in these patients.

Therefore, poor performance on tasks that assess social cognition processes could be expected in subjects with PD. Thus, the aim of this study was to compare the performance in social cognition in a clinical sample with PD and control subjects.

## Materials and Methods

### Participants

Seventeen PD patients were recruited for the study, taken from cases who were receiving treatment in the Psychiatric Unit at FLENI, Buenos Aires, Argentina. All of them had completed a thorough diagnostic process before beginning treatment and were under medication. To be included in the study, patients needed to meet the following criteria: (1) PD as a primary diagnosis. Exclusion criteria were (1) history of neurological disorder, (2) presence of psychiatric comorbidity, and (3) abnormal cognitive screening. All patients included in the study underwent a psychiatric interview using the structured clinical interview for DSM-IV personality disorders (SCID-II) to assess axis II (17, 18). A description of patients’ diagnosis and drug therapy is presented in Table 1.

**Table 1:** **Diagnostic and medication data of PD patients**.

Axis II	AD	BZD	AA	MS
Schizotypal	+		+	
Antisocial	+	+	+	+
Borderline	+			+
Borderline	+	+		
Borderline	+			
Borderline	+			
Borderline	+		+	
Borderline		+		
Histrionic	+			
Histrionic	+	+	+	
Histrionic	+	+		
Histrionic	+			
Histrionic			+	+
Histrionic	+	+		
Avoidant	+	+		
Not specific			+	+
Not specific	+	+		

*AD, antidepressant: SSRI (11), venlafaxine (1), and mirtazapine (2); BZD, benzodiazepines; AA, atypical antipsychotics: quetiapine (5) and olanzapine (1); MS, mood stabilizer*.

The patients were compared with 17 healthy control volunteer participants, included if they had no history of neurological or psychiatric disorder. The two groups were comparable with respect to age, sex ratio, and years of education. There were no significant differences between the groups on these variables (see Table 2).

**Table 2:** **Demographic and neuropsychological characteristics of both groups**.

	Patients (*N* = 17)	Controls (*N* = 17)	Statistical	*p*
Gender (F/M)	14/3	12/5	χ^2^ = 0.654	0.688
Age (years)	34 ± 15	30 ± 10	*t* = 1.01	0.32
Years of education	14.9 ± 2.8	15.4 ± 2.8	*t* = −0.49	0.628
ACE	93.4 ± 3.1	96.1 ± 1.7	*t* = −2.991	0.006
MMSE	29.4 ± 0.8	29.3 ± 1	*t* = 0.279	0.782
FAB	17.6 ± 0.6	17.6 ± 0.9	*t* = −0.33	0.974
DEX	28.1 ± 16.4	17.6 ± 8.1	*t* = 1.878	0.072
FRT	22.5 ± 02.8	24.5 ± 1.8	*t* = −2.541	0.018

*Mean ± (SD) Scores for ACE, MMSE, FAB, DEX, and BFRT*.

### Procedure

All participants signed an informed consent prior to inclusion in the study and underwent a neuropsychological assessment that included the Addenbrook’s cognitive examination (ACE) (19), MMSE (20), the frontal assessment battery (FAB) (21), and Benton facial recognition test (BFRT) (22).

### Social cognition assessment

#### Recognition of Facial Emotion

The participants were presented with 20 photographs of a woman displaying basic and complex facial emotions (23). The photographs were surrounded by a choice of the verbal labels of two emotions and the participants were asked to pick the appropriate verbal label to describe what the actress in the photograph was feeling. One point was awarded for each correct choice; the maximum score was 20.

#### Eyes Task

Photographs of the eye area of the faces of different actors displaying complex mental states were presented one at a time (24). Each pair of eyes was surrounded by a choice of four verbal labels from which the participant had to pick one to match the expression in the eyes. One point was awarded for each correct choice; the maximum score was 36.

#### Faux Pas Task

Assesses the participants’ general ability to understand and represent others’ mental states (25, 26). The faux pas task stimuli consisted of 20 stories, half of which contained a social faux pas and half of which did not. The stories were presented one at a time, each on a single page. The story text was placed in front of the participants and read aloud by the experimenter, who then asked questions about the story. To reduce the memory load of the task, participants were allowed to view the stories when they answered the question. Two points were awarded for the none faux pas stories and 6 for every faux pas story; the maximum score was 80. Each story had two control questions to make sure the subject understood the story.

#### Story Task

The stimuli consisted of 16 short stories half of which invoked ToM and the other half did not (27). The ToM stories involved either double bluff, mistakes, persuasion, or white lies and in each story the subject had to make inferences about the characters’ mental state and intentions. The administration consisted in the participants reading the story on a single page and were allowed to view this sheet when they answered the question. Two points were awarded for a full and explicitly correct answer, 1 for a partial or implicit answer, and 0 for an incorrect answer; the maximum score was 32 points.

### Data analysis

Data obtained with the SPSS 17.0 version were analyzed. Comparisons between groups for continuous variables were made through Student’s *t* distribution and correlations between these variables were performed using Spearman test. Bonferroni correction for multiple testing was applied and the thresholded for significance was *p * < 0.006. The Chi square test (χ^2^) was used to compare the gender ratio of different groups.

## Results

### General cognitive tests

No significant differences were found between the groups in MMSE, FAB, DEX, or BFRT (see Table 2).

However, there were differences between the groups in ACE where the patient group obtained significantly lower results (see Table 2).

### Social cognition

#### Recognition of Facial Emotion

**Figure 1:**
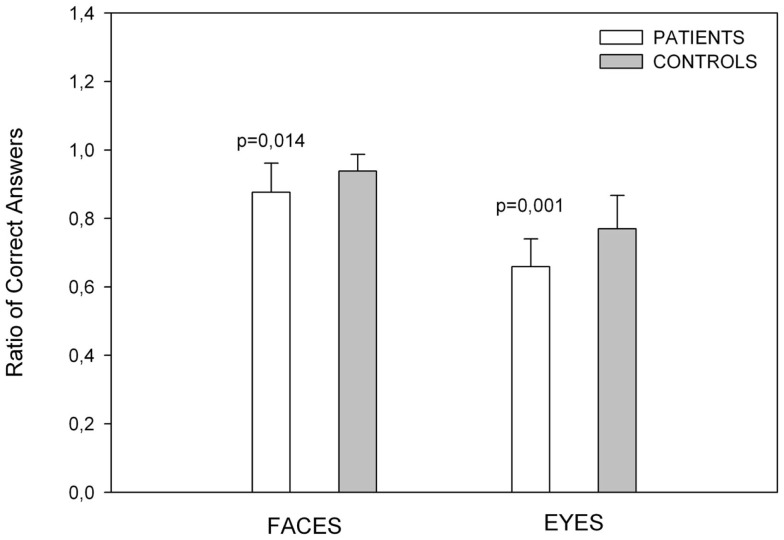
**Showing emotion recognition scores**. Control subjects had better results than patients in recognition of emotion in faces and eyes. Error bars depict standard deviations in all figures.

The scores on this test are shown in Figure 1. The patient group scored lower than the control group on the facial emotion recognition task [*t*(32) = −2.602, *p* = 0.014].

#### Eyes Task

The ratio of correct scores is showed in Figure 1. The PD group scored significantly lower than the control group [*t*(32) = −3.575, *p* = 0.001].

#### Faux Pas Task

**Figure 2:**
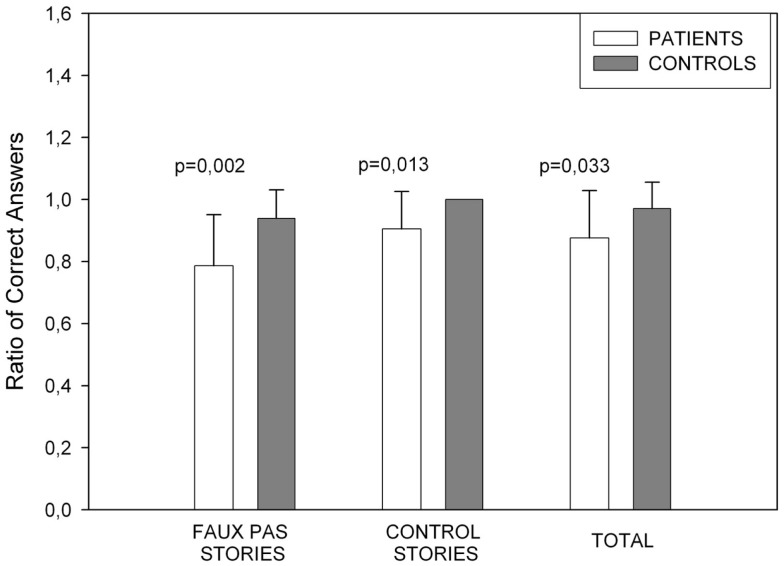
**Showing Faux pas task performance**. The patients’ performance was poorer in all Faux Pas instances. In the control stories, controls obtained the maximum score.

The measures taken were the total number of times that a participant correctly detected a faux pas when it existed in a story (hits) and the total number of times a participant correctly indicated there was not a faux pas in a story (correct rejections). A total score was obtained from the sum of both described above. The PD group performed worse than the healthy control group in the overall faux pas performance [*t*(32) = −2.227, *p* = 0.033], with a specifically a poor performance in the faux pas score [*t*(32) = −3.317, *p* = 0.002]. The number of correct rejections was also lower in the PD group [*t*(32) = −2.628, *p* = 0.013]. Figure 2 shows the Faux Pas test results.

For the control questions, we found no significant differences between groups which means that errors in this test are not attributable to failures due to experimental subject’s concentration or understanding.

#### Story Task

**Figure 3:**
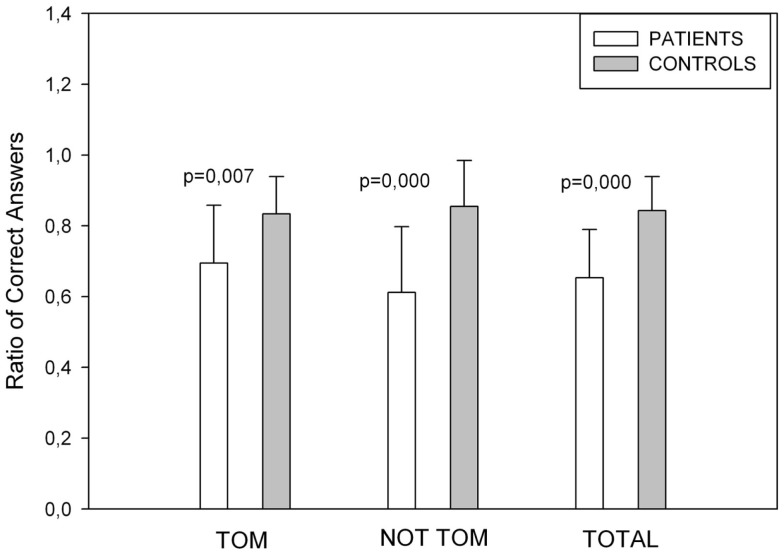
**Showing Story Task performance**. Control subjects performed better in ToM, non-ToM and in the overall score of the test.

The total numbers of correct answers for the ToM and non-ToM story conditions were computed (Figure 3). We found differences in mean scores for ToM stories [*t*(32) = −2.880, *p* = 0.007], No ToM [*t*(32) = −4.409, *p* = 0.000], and for ToM Total score [*t*(32) = −4.686, *p* = 0.000].

## Discussion

Personality disorder patients obtained lower scores relative to controls in there performance in ToM tasks and emotion recognition. Group differences cannot be explained in terms of cognitive impairment or comprehension of the tasks.

The results in a study of inmates who scored high on psychopathy (28) versus control subjects coincide with these results, as they also presented differences in recognizing faces emotions. Another study, also involving antisocial inmates (29), found that there were differences in recognizing eye emotions. The lack of accuracy in identifying emotional expression has been correlated to disinhibition, e.g., in patients with ventromedial lesions (30) and mainly to emotional dysregulation. However, recent evidence suggests that more than failing to recognize emotions there could be a tendency to negatively rate neutral or ambiguous interpersonal stimuli (31) in PD. According to the recently mentioned review, this negative bias would also apply for interpreting cognitive content in social interaction, but further research is needed to conclude to what extent this could explain the results shown in the present study. In our pilot sample, PD patients showed impaired capacity for interpreting intentions and mental states. Even though Bonferroni correction for multiple testing was used reducing statistic power to the results, the patient cohort showed a clear tendency for diminished empathy and mentalizing capacity.

The possibility of a subtle but present difficulty in social interaction needs to be explored using tools specifically designed for assessing this domain in PD (32). This issue could also help understanding the controversy in the results obtained in studies addressing PD and social cognition using the usual tasks as we did in this study (31, 33).

### Limitations

The size of the sample, the variability in the experimental subjects, which includes six subtypes of PD, as for the wide range of period under treatment and medication restrict the level of analysis of the data obtained.

Even though there were differences found between groups in the ACE performance where the PD group scored lower than the control group, all subjects were above the local cut-point for global cognitive impairment (>85) (34).

## Conclusion

In summary, the results of the sample of patients with PD showed a poor performance in tasks assessing social cognition, with an average achievement in global cognitive functioning regarding what is expected according their age and years of study. Therefore, it could be said that the PD group assessed showed a specific and subtle impairment in frontal functions regarding social interactions and empathic capacity. The extension of the results obtained in this study along with the application of structural and functional neuroimaging may contribute to the understanding of the physiological correlate underlying personality alterations.

## Conflict of Interest Statement

The authors declare that the research was conducted in the absence of any commercial or financial relationships that could be construed as a potential conflict of interest.
